# Delay in psychiatric hospitalization from the diagnosis of first-episode schizophrenia and its association with clinical outcomes and direct medical costs: a nationwide, health insurance data-based study

**DOI:** 10.1186/s12888-022-04292-5

**Published:** 2022-10-08

**Authors:** Sung Woo Joo, Harin Kim, Young Tak Jo, Soojin Ahn, Young Jae Choi, Woohyeok Choi, Soyeon Park, Jungsun Lee

**Affiliations:** 1 Department of Psychiatry, Asan Medical Center, University of Ulsan College of Medicine, Seoul, Republic of Korea; 2 Department of Psychiatry, Medical Foundation Yongin Mental Hospital, Yongin, Republic of Korea

**Keywords:** First-episode schizophrenia, Psychiatric hospitalization, Direct medical cost, Rehospitalization

## Abstract

**Background:**

Early intervention is essential for improving the prognosis in patients with first-episode schizophrenia (FES). The Mental Health Act limits involuntary hospitalization in South Korea to cases where an individual exhibits both a mental disorder and a potential for harming themselves or others, which could result in a delay in the required treatment in FES. We investigated the effect of delay in the first psychiatric hospitalization on clinical outcomes in FES.

**Methods:**

The South Korean Health Insurance Review Agency database (2012–2019) was used. We identified 15,994 patients with FES who had a record of at least one psychiatric hospitalization within 1 year from their diagnosis. A multivariate linear regression model and a generalized linear model with a gamma distribution and log link were used to examine associations between the duration from the diagnosis to the first psychiatric admission and clinical outcomes as well as direct medical costs after 2 and 5 years.

**Results:**

Within both the 2-year and the 5-year period, longer durations from the diagnosis to the first psychiatric admission were associated with an increase in the number of psychiatric hospitalizations (2-y: B = 0.003, *p* = 0.003, 5-y: B = 0.007, *p* = 0.001) and an increase in direct medical costs (total: 2-y: B = 0.005, *p* < 0.001, 5-y: B = 0.004, *p* = 0.005; inpatient care: 2-y: B = 0.005, *p* < 0.001, 5-y: B = 0.004, *p* = 0.017).

**Conclusions:**

Earlier psychiatric admission from the diagnosis is associated with a decrease in the number of psychiatric admissions as well as in direct medical costs in patients with FES.

**Supplementary Information:**

The online version contains supplementary material available at 10.1186/s12888-022-04292-5.

## Background

Early detection and intervention in schizophrenia have been emphasized in association with the improvement of long-term outcomes [1, 2]. The duration of untreated psychosis, defined as the period from the onset of psychotic symptoms to antipsychotic treatment, has been reported to be associated with treatment responses and functional recovery [3, 4]. Some countries with well-developed mental health care systmes including England, Canada, and Australia have implemented early intervention in psychosis (EIP) services in their healthcare system [5], based on evidence of the positive effects on several clinical outcomes compared with treatment as usual [6, 7]. The EIP services provide community-based multimodal treatments with the purpose of decreasing psychotic symptoms, improving social and occupational functions, and better long-term outcomes. With the tremendous economic burden of schizophrenia acknowledged globally [8], the cost-effectiveness of EIP services has also been emphasized, especially in high-income countries [9, 10].

Community-based mental health services in South Korea usually comprise the management of chronic patients with mental illnesses, the provision of social and economic support to patients and families, and education of the general public. The utilization rate of mental health facilities indicates that the South Korean mental healthcare system mostly relies on hospital-based rather than community-based treatment [11]. While some hospital-based and research-based EIP programs have been implemented, only one community-based early intervention center is currently operating in South Korea [12]. Under these circumstances, national/public and private medical institutions, not community-based mental health facilities, are mainly responsible for the detection and management of patients with early psychosis. Owing to the limited resources available for patients at medical institutions, the comprehensive treatment programs included in EIP services cannot be provided in outpatient settings. An interview-based study reported that a large proportion of patients in South Korea spent only a few minutes with their clinician during their visit to an outpatient department [13]. Thus, given the lack of community-based EIP services in South Korea and the importance of a multimodal, multidisciplinary team approach in early psychosis, inpatient treatment would be an alternative for providing the intensive care required during the critical early period of schizophrenia.

In 2016, the Mental Health Promotion and Welfare Act in South Korea was revised to limit involuntary admission to patients exhibiting both a mental illness and a risk of likely self-harm or of harming others [14]. Despite provisional expectations for promoting patients’ human rights, it has been argued that the necessary treatment may be delayed owing to the narrowed conditions for involuntary admission, thus resulting in a worsening prognosis [15]. According to the Mental Health Promotion and Welfare Act, it is mandatory to receive the consent of the patient’s legal guardian for involuntary admission, which has also been criticized in that the responsibility for medical or legal decisions is passed on to family members who have to live with the patients [16]. Previous studies have reported on the experiences of family caregivers who had to decide about involuntary admissions and felt that too much responsibility was placed on them even though they had not been fully informed about treatment decisions regarding inpatient care [17, 18]. Inpatient care of a patient with schizophrenia against their will, even if they have poor insight and prominent psychotic symptoms, is not allowed unless there is actual evidence on the risk of self-injury or injury to others; this means that the decision largely depends on patients who are acutely psychotic and can be expected to experience difficulty making the right judgment.

### Aims of the study

In this study, we investigated clinical outcomes according to the duration from the diagnosis to the first psychiatric admission in first-episode schizophrenia (FES) patients, using claims data from the Health Insurance Review and Assessment (HIRA) database in South Korea. We hypothesized that in FES patients who require inpatient care after their diagnosis, a delay in the first psychiatric hospitalization would be associated with poor outcomes in terms of the risk of treatment discontinuation and psychiatric rehospitalization, as well as with increased direct medical costs. Additionally, we investigated the extent to which the patients were undergoing necessary antipsychotic treatment before their first psychiatric hospitalization, based on abundant evidence emphasizing the importance of treatment compliance in the early phase of the disease in association with long-term clinical outcomes in schizophrenia [19].

## Methods

### Data source

We retrieved the claims data from between 2010 and 2019 from the HIRA database. The details of the HIRA database are described elsewhere [20]. Briefly, the HIRA claims data are created during the process of reimbursing claims for medical services in the National Health Insurance System (NHIS) in South Korea. All South Koreans are obligated to register in the NHIS, under the term of universal health coverage. Since 2009, the HIRA data are publicly available for research purposes. Information on healthcare services provided to beneficiaries is included in the HIRA database, including examinations, procedures, surgeries, drug prescriptions, as well as the patients’ sociodemographic characteristics.

### Identification of incident patients with schizophrenia

Incident patients with schizophrenia were defined according to the following inclusion criteria: 1) the main diagnostic code F20 (schizophrenia) was recorded at least twice for outpatients or once for inpatients during the total observation period; 2) the diagnosis of schizophrenia was determined as the first registration of the main diagnostic code F20-29 (schizophrenia, schizotypal, delusional, and other non-mood psychotic disorders) in the HIRA database and the age at diagnosis was between 18 and 45 years; 3) the patient was not diagnosed with the main diagnostic code F20-29 for at least 2 years before the initiation of the observation period; 4) the diagnostic code F20-29 was included at the last psychiatric visit, which was identified by the main diagnostic code F00-99 (mental and behavioral disorders); 5) more than 30 days of antipsychotic treatment were recorded during the total observation period; and 6) the observation period was longer than 1 year. Based on the above criteria, a total of 33,117 FES patients were identified. The present study was approved by the Institutional Review Board of Asan Medical Center (IRB No. 2021–0556). The requirement for informed consent was waived owing to the use of anonymous and de-identified data.

### Definitions of event outcomes and direct medical cost

We created admission episodes according to a previous study [21], because the claims data for admission, the duration of which was more than 1 month, was generated separately every month. An admission was classified as psychiatric if it was general medical or psychiatric and accompanied by the main diagnostic code F00-99. Rehospitalization within 30 days from discharge following a prior admission was regarded as the continuation of the prior admission, based on a previous study [22], because it was considered an indication of inadequate treatment during the former admission. Treatment discontinuation was determined based on a gap of more than 30 days between antipsychotic treatments. If an antipsychotic prescription was not recorded within 30 days from the expected date of the next prescription, this was considered a treatment discontinuation. Supplementary Table 1 shows a list of antipsychotic drugs, including those assessed in the current study. The HIRA general research data contains cost-related information, such as patient out-of-pocket costs and payer costs, as well as other sociodemographic information including age, sex, type of insurance, and de-identified beneficiary and provider identification. We calculated the direct medical costs using the total cost information (RVD_RPE_TAMT_AMT) provided in the general information file. The direct medical costs comprise inpatient and outpatient care costs as well as pharmacy costs, but exclude costs that are not covered by the insurance provider. The costs for inpatient care were differentiated by the code indicating the type of claims data (i.e., FOM_TP_CD of 21 or 101), and the costs for psychiatric disorders were determined by the main diagnostic code F00-99. The costs for all medical disorders were defined as the sum of the direct medical costs for any healthcare services during a specific period. The prevalence approach was used for the direct medical costs, which were expressed in Korean Won (KRW).

We selected 21,902 FES patients with at least one recorded psychiatric admission during the total observation period. To evaluate treatment compliance in the period between the diagnosis and the first psychiatric admission, we calculated the medication possession ratio (MPR), which is the ratio of the sum of the days of antipsychotic prescriptions to all days in a specific period. Previous studies using claims databases have adopted the MPR as a measurement of treatment adherence in schizophrenia patients; an MPR below 0.8 is usually considered to represent poor treatment adherence [23, 24]. Based on previous studies [25, 26] showing a high discontinuation rate of antipsychotic treatment within 1 year from treatment initiation in first-episode psychosis (FEP), we selected 15,994 FES patients for the current study for whom the duration from the diagnoses to the first psychiatric admission amounted to less than 1 year.

### Statistical analysis

We investigated clinical outcomes and direct medical costs within periods of 2 and 5 years from the first psychiatric admission. The 2- and 5-year periods were determined according to the commonly used definitions for early schizophrenia, which include an illness duration of less than 2 or less than 5 years [27]. The clinical outcomes included the duration of the first psychiatric admission, number of treatment discontinuations, and psychiatric admissions. We calculated the direct medical costs for inpatient care and the total direct medical costs for both psychiatric disorders and all medical disorders. Although our main interest in the current study was to examine the effect of a delay in the first psychiatric admission on clinical outcomes and direct medical costs, we believe that the MPR for the period between the diagnosis and the first psychiatric admission also contributes to these outcomes. Therefore, we investigated a change in the MPR according to the duration from the diagnosis to the first psychiatric admission. We performed multivariate linear regression analyses on the relationship between the duration from the diagnosis to the first psychiatric admission and the clinical outcomes. A generalized linear model with a gamma distribution and log link was used to examine the association with direct medical costs. The age at diagnosis, sex, calendar year of the diagnosis, type of hospital that made the diagnosis, and MPR were included in the analyses as covariates. All statistical analyses were performed using R software ver. 3.5.1 (R Development Core Team, Vienna, Austria). Statistical significance was determined using an alpha value of 0.05.

## Results

### Demographic and clinical characteristics of the study population

Table 1 presents the demographic and clinical characteristics of the 15,994 FES patients. The mean (SD) age at diagnosis was 30.8 (7.8) years, and men made up 49.8% (*n* = 7,967) of all patients. The largest number of patients were diagnosed with FES at a hospital (*n* = 6,369, 39.8%). The average (SD) duration of the observation period was 5.3 (1.7) years; additionally, the mean (SD) numbers of psychiatric admissions and treatment discontinuations during the total observation period were 2.3 (1.8) and 2.8 (3.4). The psychiatric admissions occurred at an average (SD) of 5.4 (10.5) weeks from the diagnosis of FES.

**Table 1:** Demographic and clinical characteristics of patients with first-episode schizophrenia

Variable	Value (*N* = 15,994)
Age at diagnosis, mean (SD), years	30.8 (7.8)
Age group, n (%)	
18–19	1084 (6.8)
20–24	3297 (20.6)
25–29	2927 (18.3)
30–34	3002 (18.8)
35–39	2763 (17.3)
40–45	2921 (18.3)
Sex, n (%)	
Male	7967 (49.8)
Type of hospital making the diagnosis, n (%)	
Tertiary hospital	2953 (18.5)
General hospital	2916 (18.2)
Hospital	6369 (39.8)
Nursing hospital	657 (4.1)
Clinic	3099 (19.4)
Calendar year of the diagnosis, n (%)	
2012	3578 (22.4)
2013	2979 (18.6)
2014	2714 (17.0)
2015	2400 (15.0)
2016	2232 (14.0)
2017	2091 (13.1)
Duration of the observation period, mean (SD), years	5.3 (1.7)
Duration from diagnosis to psychiatric admission, mean (SD), weeks	5.4 (10.5)
Number of psychiatric admissions ^a^, mean (SD)	2.3 (1.8)
Number of treatment discontinuations ^a^, mean (SD)	2.8 (3.4)

^a^ calculated for the total observation period

**Fig. 1:**
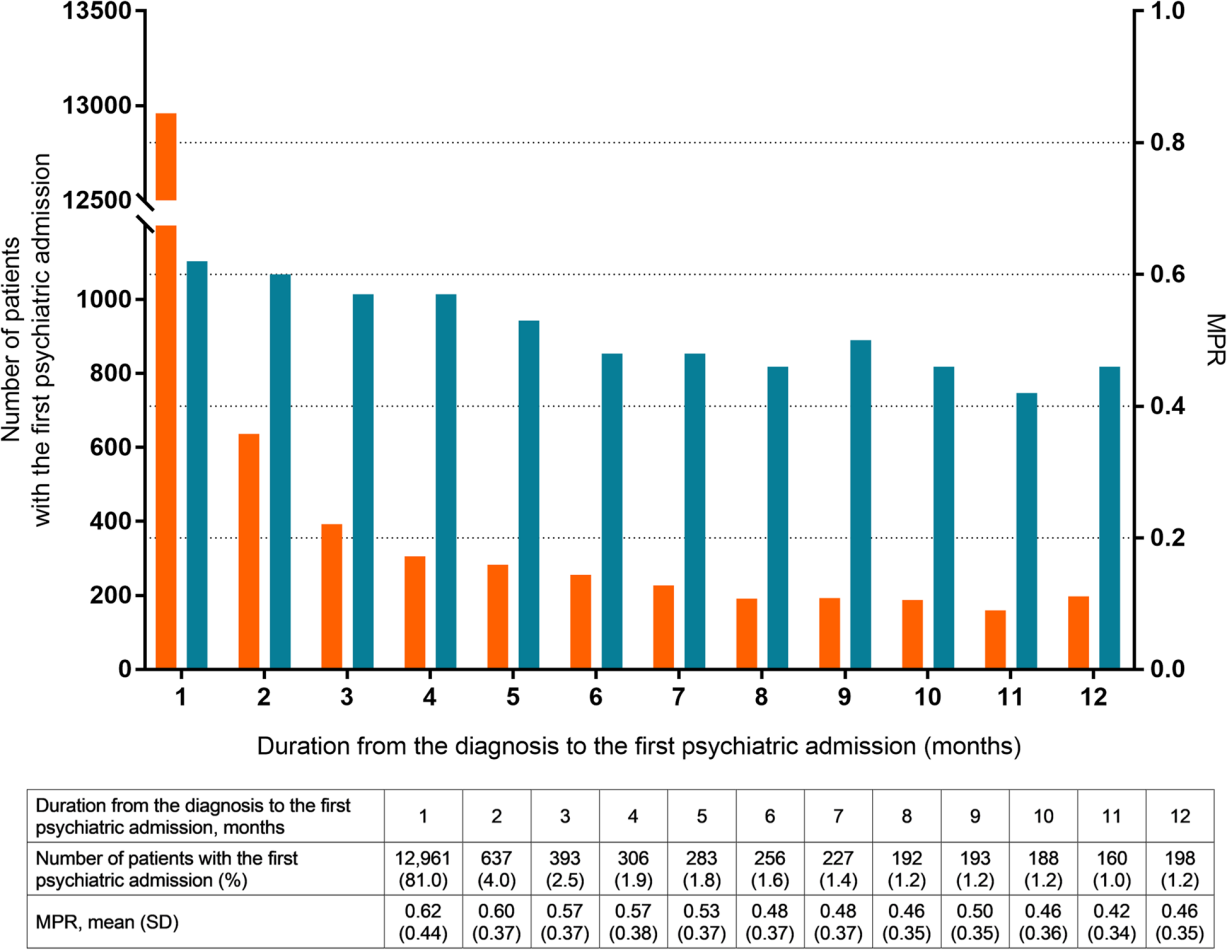
Number of patients with first psychiatric admissions and medication possession ratio according to the duration from diagnosis to first psychiatric admission. The orange bars indicate the numbers of patients with first psychiatric admissions, and the blue bars denote the mean medication possession ratios

Figure 1 shows the number of patients with a first psychiatric admission and the MPR according to the duration from the diagnosis to the first psychiatric admission. A substantial proportion of patients (*n* = 12,961, 81.0%) had their first psychiatric admission within 1 month from their diagnosis. A decreasing trend was observed in the MPR as the duration from the diagnosis to the psychiatric admission increased.

### Associations with clinical outcomes and direct medical costs

#### Duration of first psychiatric admission and number of treatment discontinuations and psychiatric admissions

The results of the multivariate linear regression analyses on the associations between the duration of the first psychiatric admission and the number of treatment discontinuations and psychiatric admissions are presented in Tables 2 and 3. The MPR showed a negative association with the number of treatment discontinuations in the 2-year (B = -0.320, t = -8.816, *p* < 0.001) and 5-year (B = -0.607, t = -6.758, *p* < 0.001) periods. The duration from the diagnosis to the psychiatric admission was associated with an increase in the number of psychiatric admissions in the 2 two-year (B = 0.003, t = 3.013, *p* = 0.003) and 5-year (B = 0.007, t = 3.284, *p* = 0.001) periods.

**Table 2:** Associations with clinical outcomes in the 2-year period following the first psychiatric hospitalization^a^

Variable	Duration of the first psychiatric hospitalization (days)			Number of treatment discontinuations			Number of psychiatric hospitalizations		
	B	t	p	B	t	p	B	t	p
MPR^b^	-9.035	-0.987	0.324	-0.320	-8.816	< 0.001	0.030	0.930	0.352
Duration from diagnosis to first psychiatric hospitalization (weeks)	0.028	0.108	0.914	-0.001	-0.740	0.459	0.003	3.013	0.003

*MPR* medication possession ratio

^a^ Multivariate linear regression analysis was performed with age at diagnosis, sex, type of hospital making the diagnosis, and calendar year of the diagnosis as covariates

^b^ Calculated using the antipsychotic prescriptions during the period from the diagnosis to the first psychiatric hospitalization

**Table 3:** Associations with clinical outcomes in the 5-year period following the first psychiatric hospitalization^a^

Variable	Duration of the first psychiatric hospitalization (days)			Number of treatment discontinuations			Number of psychiatric hospitalizations		
	B	t	p	B	t	p	B	t	p
MPR^b^	-7.427	-0.526	0.599	-0.607	-6.758	< 0.001	-0.058	-0.765	0.444
Duration from diagnosis to first psychiatric admission (weeks)	-0.268	-0.676	0.499	0.001	0.487	0.626	0.007	3.284	0.001

*MPR* medication possession ratio

^a^ Multivariate linear regression analysis was performed with age at diagnosis, sex, type of hospital making the diagnosis, and calendar year of the diagnosis as covariates

^b^ Calculated using the antipsychotic prescriptions during the period from the diagnosis to the first psychiatric hospitalization

#### Direct medical costs for psychiatric illnesses and all medical disorders

The associations with direct medical costs are presented in Tables 4 and 5. In the 2-year period, the duration from the diagnosis to the first psychiatric admission was associated with an increase in the costs for inpatient care (psychiatric disorders: B = 0.005, t = 4.180, *p* < 0.001; all medical disorders: B = 0.004, t = 3.804, *p* < 0.001) and the total costs (psychiatric disorders: B = 0.005, t = 5.226. *p* < 0.001; all medical disorders: B = 0.005, t = 5.038, *p* < 0.001). In the 5-year period, there was a positive relationship between the duration from the diagnosis to the first psychiatric admission and the costs for inpatient care (psychiatric disorders: B = 0.004, t = 2.386, *p* = 0.017; all medical disorders: B = 0.004, t = 2.451, *p* = 0.014) and the total costs (psychiatric disorders: B = 0.004, t = 2.808, *p* = 0.005; all medical disorders: B = 0.003, t = 2.938, *p* = 0.003).

**Table 4:** Associations with direct medical costs in the 2-year period following the first psychiatric hospitalization^a^

	Psychiatric disorders				All medical disorders			
	Total		Inpatient care		Total		Inpatient care	
Variable	Exp(coef) (95% CI)	t (*p* value)	Exp(coef) (95% CI)	t (*p* value)	Exp(coef) (95% CI)	t (*p* value)	Exp(coef) (95% CI)	t (*p* value)
Intercept (KRW)	7,329,338 (6,407,938–8,389,772)	231.138 (< 0.001)	6,882,090 (5,793,589–8,186,606)	180.307 (< 0.001)	7,136,430 (6,252,659–8,151,407)	233.055 (< 0.001)	6,534,485 (5,506,106–7,765,616)	180.152 (< 0.001)
MPR^b^	0.956 (0.897–1.018)	-1.387 (0.166)	0.959 (0.886–1.038)	-1.030 (0.303)	1.005 (0.944–1.070)	0.146 (0.884)	0.971 (0.897–1.051)	-0.726 (0.468)
Duration from diagnosis to first psychiatric admission (weeks)	1.005 (1.003–1.007)	5.226 (< 0.001)	1.005 (1.002–1.007)	4.180 (< 0.001)	1.005 (1.003–1.006)	5.038 (< 0.001)	1.004 (1.002–1.007)	3.804 (< 0.001)

*KRW* Korean won, *MPR* medication possession ratio

^a^ Generalized linear model with a gamma distribution and log link was used with covariates of age at diagnosis, sex, type of hospital making the diagnosis, and calendar year of the diagnosis

^b^ Calculated using the antipsychotic prescriptions during the period from the diagnosis to the first psychiatric hospitalization

**Table 5:** Associations with direct medical costs in the 5-year period following the first psychiatric hospitalization^a^

	Psychiatric disorders				All medical disorders			
	Total		Inpatient care		Total		Inpatient care	
Variable	Exp(coef) (95%CI)	t (*p* value)	Exp(coef) (95%CI)	t (*p* value)	Exp(coef) (95%CI)	t (*p* value)	Exp(coef) (95%CI)	t (*p* value)
Intercept (KRW)	15,984,707 (13,381,200–19,122,894)	179.48 (< 0.001)	11,944,384 (9,353,789–15,299,123)	128.224 (< 0.001)	16,207,654 (13,741,233–19,141,432)	192.677 (< 0.001)	11,444,542 (9,010,832–14,577,330)	130.059 (< 0.001)
MPR^b^	0.985 (0.902–1.074)	-0.345 (0.730)	0.968 (0.860–1.090)	-0.527 (0.599)	1.051 (0.968–1.140)	1.176 (0.240)	0.993 (0.883–1.115)	-0.123 (0.902)
Duration from diagnosis to first psychiatric admission (weeks)	1.004 (1.000–1.006)	2.808 (0.005)	1.004 (1.001–1.008)	2.386 (0.017)	1.003 (1.001–1.006)	2.938 (0.003)	1.004 (1.001–1.008)	2.451 (0.014)

*KRW* Korean won, *MPR* medication possession ratio

^a^ Generalized linear model with a gamma distribution and log link was used with covariates of age at diagnosis, sex, type of hospital making the diagnosis, and calendar year of the diagnosis

^b^ Calculated using the antipsychotic prescriptions during the period from the diagnosis to the first psychiatric hospitalization

## Discussion

In this study, we investigated the association between the duration from the diagnosis to the first psychiatric admission and clinical outcomes and direct medical costs in the 2 and 5 years after a patient’s first psychiatric admission, using a large-scale nationwide health insurance database. Of the 15,994 FES patients who underwent their first psychiatric admission within 1 year from the diagnosis, a majority (81.0%) were admitted to a psychiatric hospital within 1 month. The treatment compliance, measured using the MPR, exhibited a decreasing trend as the duration from the diagnosis to the first psychiatric admission increased. Moreover, the MPR was negatively associated with the number of treatment discontinuations within the 2 and 5 years. Longer durations from the diagnosis to the first psychiatric admission were associated with an increase in the number of psychiatric admissions and an increase in direct medical costs for both psychiatric disorders and all medical disorders within the 2 and 5 years.

We identified a total of 33,117 FES patients, approximately 66.1% (21,902) of whom underwent psychiatric admission at least once during the total observation period. By applying a 1-year criterion to exclude patients who were admitted to a psychiatric hospital owing to relapse, a total of 15,994 (48.3%) FES patients who underwent psychiatric admission owing to their first psychotic episode were identified. Recent studies employing health administrative data revealed that one in three incident patients with non-affective psychosis undergoes their first psychiatric admission within 2 years after the onset of psychosis and that 81% of patients are involuntarily admitted to a psychiatric hospital [28, 29]. The authors also found that the involvement of the family physician in the diagnosis represents a protective factor and can reduce the risk of hospitalization. Our results reveal a higher rate of psychiatric admission than that reported in previous studies. This may reflect the lack of community-based mental health and EIP services in South Korea [11], which emphasizes the necessity to further efforts to decrease the rate of psychiatric hospitalization in FES patients.

We observed a decreasing trend in the MPR according to the increase in the duration from the diagnosis to the first psychiatric hospitalization, indicating poor compliance with antipsychotic treatment in patients with longer durations to their first psychiatric admission. A previous study revealed that 45.1% of FEP patients are nonadherent to antipsychotic treatment and more likely to refuse medication at the first offer of treatment [30]. A recent study reported a 75.4% rate of nonadherence over 2 years of antipsychotic treatment in FEP patients [31]. Together with the protection and promotion of patients’ rights regarding treatment decisions, it is necessary to improve their treatment compliance after the onset of psychosis, based on evidence supporting the negative impact of the duration of untreated psychosis on prognoses [4].

The MPR was negatively associated with the number of treatment discontinuations within 2 and 5 years in our data, suggesting that treatment compliance in the early phase is closely related to that in the later period. A lack of insight has been identified as a key driver of nonadherence to medications in schizophrenia patients [32]. In a prospective study on the longitudinal changes in insight in FEP, up to 50% of patients exhibited a lack of insight at the baseline; additionally, insight and level of education were predictive of treatment adherence [33]. Our results indicate that further strategies to improve patients’ insight are needed, from the early phase, along with the multifactorial risk factors associated with medication nonadherence [34].

Longer durations from the diagnosis to the first psychiatric hospitalization were associated with an increase in the number of psychiatric admissions and an increase in direct medical costs for psychiatric disorders and all medical disorders in our sample. In previous studies, psychiatric rehospitalization has been used as a marker for relapse in FEP [35, 36]. Our results indicate that delays in the first psychiatric admission are associated with a higher risk of relapse in FES. Several risk factors associated with relapse in FEP have been reported, including persistent substance use, unemployment, and poor premorbid adjustment [37, 38]. While it could be postulated that delays in the first psychiatric admission are intertangled with the other known risk factors for relapse, thus cumulatively contributing to the increased rate of psychiatric rehospitalization, it is also possible that psychiatric rehospitalization occurs for reasons other than relapse. Further studies are needed to elaborate on the contributing effect of delays in first psychiatric admissions on the increased number of psychiatric rehospitalizations.

EIP programs have been proven effective in reducing medical costs compared with treatment as usual [9, 39]. We found that earlier psychiatric admission after the diagnosis was associated with reduced direct medical costs for psychiatric disorders and all medical disorders. Given the circumstances regarding poor community-based mental health services and a lack of time allowed for patients in outpatient settings in South Korea, inpatient care can be beneficial in terms of more intensive care and comprehensive treatments. While the variability in the quality of inpatient services across medical institutions should be considered, we addressed this issue by including the covariate “type of hospital” in our analyses. Even when admission is voluntary, a decision about hospitalization is based on a discussion with the family caregiver of the patient, which partially guarantees family involvement in the treatment. We observed an increase in direct medical costs for all medical disorders in the 2- and 5-year periods, but this should be interpreted as an exploratory finding, since other factors that were not considered in the present study could contribute to the change in direct medical costs; the increase in direct medical costs for all medical disorders could thus be inflated or deflated. Further studies are required to investigate this issue in a more detailed manner.

We investigated the effect of delays in first psychiatric admissions after the diagnosis on clinical outcomes and direct medical costs in FES patients. We obtained a large sample size by utilizing a nationwide health insurance database, which is one of the strengths of the current study. Some limitations of this study should, however, be considered in the interpretation of our results. First, the current study was based on claims data, in which some clinical information was missing. Although we applied strict criteria for selecting FES patients, the definition of FES remains somewhat arbitrary. Second, using a criterion of 1 year from the diagnosis, we excluded patients who underwent psychiatric admissions owing to relapse. Although this was based on previous studies reporting a high rate of treatment discontinuation within 1 year after the initiation of antipsychotic treatment in FEP patients, there is a possibility that not all patients who relapsed were excluded and not all patients in their first episode were included. Third, we only examined the direct medical costs associated with delays in the first psychiatric admission, and existing evidence suggests that direct medical costs are only a small subset of the total costs associated with schizophrenia [40, 41]. Our future studies will have a broader scope to also explore the impact of delayed first psychiatric admissions on indirect costs, such as unemployment, workplace productivity loss, and caregiving costs.

## Conclusion

We investigated the association between the duration from the diagnosis to the first psychiatric admission and clinical outcomes and direct medical costs in FES patients. A decreasing trend in the MPR was observed as the delay in the first psychiatric admission was prolonged. Furthermore, The MPR was negatively associated with the number of treatment discontinuations within the 2- and 5- year periods, while the direct medical costs for psychiatric disorders and all medical disorders positively correlated with the duration from the diagnosis to the first psychiatric admission. We suggest that early psychiatric admission may have a beneficial effect on a number of clinical outcomes in FES patients. However, these findings should be interpreted in the context of the lack of community-based mental health services in South Korea. The current results emphasize the importance of early-phase treatment in improving the long-term outcomes of FES patients rather than focusing on psychiatric hospitalization alone.

## Supplementary Information

**Additional file 1:** **Supplementary Table 1.**List of typical and atypical antipsychotics.

## Data Availability

The datasets used in this study are available from the Health Insurance Review and Assessment service on reasonable request. Further details on the utilization of the HIRA database are presented elsewhere (http://opendata.hira.or.kr).

## Abbreviations

FES: First-episode schizophrenia
FEP: First-episode psychosis
HIRA: Health Insurance Review and Assessment
MPR: Medication possession ratio
