# Supplementary material for: Delay in psychiatric hospitalization from the diagnosis of first-episode schizophrenia and its association with clinical outcomes and direct medical costs: a nationwide, health insurance data-based study
Source: BMC Psychiatry. 2022 Oct 8;22:636. doi: 10.1186/s12888-022-04292-5 (PMC9548117; doi:10.1186/s12888-022-04292-5)
Supplement: Supplementary file 1 — Additional file 1: Supplementary Table 1.List of typical and atypical antipsychotics. [file 12888_2022_4292_MOESM1_ESM.docx]

**Supplementary Table 1.** List of typical and atypical antipsychotics

| Typical antipsychotics | | Atypical antipsychotics | |
| --- | --- | --- | --- |
| Oral | LAI | Oral | LAI |
| Bromperidol | LAI Haloperidol | Amisulpride | LAI Aripiprazole |
| Chlorpromazine |  | Aripiprazole | LAI Paliperidone^a^ |
| Chlorprothixene |  | Blonanserin | LAI Risperidone |
| Haloperidol |  | Clozapine |  |
| Levomepromazine |  | Olanzapine |  |
| Molindone |  | Paliperidone |  |
| Nemonapride |  | Quetiapine |  |
| Perphenazine |  | Risperidone |  |
| Pimozide |  | Sulpiride |  |
| Thiothixene |  | Ziprasidone |  |
| Trifluoperazine |  | Zotepine |  |

LAI: long-acting injectable

^a^ includes paliperidone palmitate monthly (Invega Sustenna) and 3-month injections (Invega Trinza)
